# Migration of Diethyl-Hexyl-Phthalate from Plastic Containers and Oak Casks to Tequila During Long-Term Storage and Aging

**DOI:** 10.3390/foods15081380

**Published:** 2026-04-15

**Authors:** Jose Tomas Ornelas-Salas, Oscar F. Caselin-Garcia, Jose de Jesus Gomez-Guzman, Daniel Alcala-Sanchez, Juan Carlos Tapia-Picazo, Antonio De Leon-Rodríguez

**Affiliations:** 1 Departamento de Ingeniería Química, Tecnológico Nacional de México-Instituto Tecnológico de Aguascalientes, Av. Adolfo López Mateos 1801, Ote. Fracc. Bona Gens, Ags., Aguascalientes C.P. 20256, Mexico; 2 Maestría en Procesos del Tequila, Universidad Autónoma de Guadalajara, Av. Patria 1201, Lomas del Valle 3ª Sección, Jal., Zapopan C.P. 45129, Mexico; 3 División de Biología Molecular, Instituto Potosino de Investigación Científica y Tecnológica, Camino a la Presa San José 2055, S.L.P., San Luis Potosí C.P. 78216, Mexico

**Keywords:** tequila, alcoholic beverages, phthalate, DEHP, endocrine disruptor

## Abstract

Tequila is frequently stored or aged in polymer containers and oak casks, which can enable the migration of phthalates such as di-(2-ethylhexyl) phthalate (DEHP), an endocrine-disrupting chemical. We quantified DEHP in tequila (55% ethanol) stored in LDPE tanks, HDPE jerry cans, PET carboys, and French oak casks with and without thermal treatment during long-term storage/aging (up to 18 and 11 months, respectively). Monthly samples were extracted and analyzed by GC–MS. Migration kinetics were evaluated using empirical exponential/sigmoidal models and an analytical solution of Fick’s second law for a semi-infinite slab. In plastics, DEHP increased nonlinearly and was best described by a modified Gompertz model, exhibiting a lag phase up to 42 days (~month 2), maximum transfer rates (Rmax) up to 0.82 µg L^−1^ day^−1^, and late-time concentrations near 120 µg L^−1^. The non-toasted oak cask previously used for wine showed exponential behavior, reaching ~185 µg L^−1^ and fitting the Minchev–Minkov model, whereas the toasted cask showed minimal transfer. Although concentrations remained below a reference safety limit (1500 µg kg^−1^), the results indicate that food-contact plastics and commonly used oak casks are not risk-free under prolonged contact, supporting model-based forecasting for quality control.

## 1. Introduction

Tequila is an alcoholic beverage protected under the Denomination of Origin, produced exclusively within designated regions of Mexico, and regulated by the Official Mexican Standard NOM-006-SCFI-201211 [1]. Its production involves the fermentation of carbohydrates derived from the hydrolysis of the inulin present in *Agave tequilana* Weber var. *azul*, followed by distillation [1,2]. During post-distillation, tequila may be diluted to adjust its alcohol content, aged in oak barrels, and subsequently bottled in either glass or polyethylene terephthalate (PET) containers [1].

Although stainless steel tanks are preferential storage vessels, small and medium-sized tequila producers frequently use plastic containers due to their cost-effectiveness and ease of handling. Table 1 summarizes the primary types of plastic vessels utilized in the tequila industry, which may remain in contact with the beverage for up to two years or more. Even though these plastics are categorized as food-grade, they can contain trace amounts of phthalates [3,4].

Among the phthalates, di-(2-ethylhexyl) phthalate (DEHP) is one of the most used due to its effectiveness as a plasticizer, accounting for nearly 50% of all plasticizers employed in polymer manufacturing [5]. However, DEHP is classified as an endocrine disruptor with documented carcinogenic, cytotoxic, and anti-androgenic activities [6,7,8,9]. Given its adverse health effects, international regulations have established permissible limits for phthalates in food products, including beverages [10]. Table 2 presents maximum allowable concentrations for various phthalates, including DEHP, in non-fatty foods and distilled alcoholic beverages according to international standards.

Previous studies have reported the migration of phthalates into alcoholic beverages due to contact with plastic materials or equipment incorporating polymeric seals or coatings [14,15,16,17,18]. Specifically for tequila, earlier studies revealed the presence of DEHP both in commercially bottled beverages and at different stages of the production process, particularly when using plastic materials or containers [14,15]. Moreover, oak barrel aging, especially barrels previously used to age other spirits or wines, can introduce residual phthalates present in the wood into the tequila [16].

Although DEHP contamination in tequila has been identified, there are relatively few detailed studies addressing the kinetics of DEHP migration from plastic containers and oak barrels. The absence of comprehensive kinetic data makes it challenging to precisely estimate DEHP levels transferred to tequila over prolonged storage periods, and thus it remains unclear which storage conditions pose the greatest risks.

This study examines the migration of DEHP from various plastic containers (large tanks, barrels, and carboys) and oak barrels (with and without thermal treatment) into tequila. To elucidate the transfer mechanisms, we employed comparative evaluations of exponential and sigmoidal mass-transfer models, alongside an analytical model based on Fick’s diffusion laws (semi-infinite slab). Considering tequila’s economic and cultural significance in Mexico and the associated health risks of phthalate ingestion, this research aims to provide a predictive tool for the tequila industry to (1) select appropriate plastic containers and recycled oak barrels, (2) anticipate phthalate concentration increases during prolonged storage and aging, and (3) establish robust quality control strategies to ensure product safety. Furthermore, we examined the suitability of various models with respect to material porosity and composition, phthalate solubility in hydroalcoholic solutions, and potential long-term exposure scenarios. This integrated approach not only enriches tequila-specific literature but also establishes a foundation for applying migration kinetic models to other alcoholic beverages or food systems susceptible to phthalate contamination.

The novelty of this research lies in the fact that, unlike previous studies that often address short-term migration or a single packaging category, we provide a long-term, side-by-side assessment of DEHP transfer into tequila (55% ethanol) from industrially relevant plastic containers (LDPE, HDPE, PET) and French oak casks with contrasted thermal treatment histories. We further connect surface/microstructural features (SEM) and material chemistry (FTIR) to the observed kinetics, and benchmark multiple empirical kinetic models against an analytical solution of Fick’s second law for a semi-infinite slab to support conservative, model-based forecasting for quality control and risk management. Note that ATR–FTIR is primarily used here to confirm the polymer/wood chemical fingerprint; DEHP at mg·kg^−1^ levels may be below ATR–FTIR detectability and its ester bands can overlap with the polymer matrix. Therefore, DEHP presence and levels were confirmed quantitatively by GC–MS.

## 2. Materials and Methods

Packaging metadata and polymer-DEHP determination: For each packaging item, we recorded the approximate container mass and measured representative wall thicknesses with a caliper at several locations (reported as an approximate range because thickness varies by geometry). DEHP in the packaging polymer was determined by sampling polymer pieces from non-printed areas (labels/inks excluded), solvent extraction, and GC–MS quantification using the same calibration strategy described for tequila. All containers were marketed as food-contact materials; we also indicated whether each item was new or previously used at the start of the experiment, as prior use and recycled content can influence background plasticizer levels. GC–MS acquisition and QA/QC: The MS acquisition mode (SIM where applicable) and injector settings (including inlet temperature) have been specified for full reproducibility. LOD/LOQ were calculated using a signal-to-noise approach and calibration-based criteria as detailed below. To control for ubiquitous phthalate background, solvent blanks, procedural blanks, and glassware controls were run routinely; baseline DEHP in the starting tequila (t = 0) was treated as the initial condition for kinetic fitting.

### 2.1. Chemicals and Materials

Purity/grade: The DEHP analytical standard was of high purity (supplier certificate), and all solvents used for extraction and GC–MS analysis were analytical grade (or higher) as received from the supplier.

All reagents and solvents used were of analytical grade. DEHP standard (purity ≥ 99.5%) was purchased from Sigma-Aldrich (St. Louis, MO, USA). Dichloromethane (DCM), hexane and ethanol were obtained from JT Baker (Mexico). The tequila used in this study was 100% agave “*blanco*” tequila with an ethanol content of 55% (*v*/*v*), provided by a certified distillery in the Tequila Valley region (Tequila, Jal., Mexico). The tequila was free of added sugar and was stored in stainless steel tanks prior to experimentation. For the study, the three types of plastic containers shown in Table 3 were selected.

Two 225 L French oak barrels were also used. One had previously been employed in wine aging (non-toasted), and the other had been used for whisky aging (toasted). Both barrels were purchased from cooperages in Mexico and inspected prior to use.

### 2.2. Container Characterization

The plastic containers were analyzed via Scanning Electron Microscopy (SEM) to evaluate their surface morphology. Samples (2 cm × 2 cm) were cut from each plastic surface, fixed to aluminum stubs with carbon tape, and sputter-coated with gold. Micrographs were taken using a JEOL JSM-6510 microscope (JEOL Ltd., Tokyo, Japan) operated at 20 kV.

Wood morphology: For oak, SEM highlights intrinsic porosity (vessel elements, rays) and surface changes associated with thermal treatment (toasting), which can modify permeability and accessible pathways; these features are discussed in the context of the contrasting migration observed between the non-toasted and toasted casks.

SEM rationale: SEM was used to qualitatively assess surface topography (roughness, fissures, microvoids) and, for wood, pore/vessel features that can increase effective interfacial area and create preferential pathways for sorption and diffusion, helping interpret kinetic differences among materials.

Fourier Transform Infrared Spectroscopy (FTIR) was used to confirm the polymer composition of each container. Spectra were recorded in the range of 4000–400 cm^−1^ using a Bruker Alpha Platinum ATR spectrometer (Bruker Optics, Ettlingen, Germany), and the functional groups corresponding to LDPE, HDPE, and PET were identified based on reference spectra.

In addition, ATR–FTIR spectra were collected for both oak casks (non-toasted and toasted) to characterize the main wood components and to support the interpretation of the chemical differences observed in the aging materials. Measurements were performed using the same instrument and acquisition settings as described above; spectra were recorded from 4000 to 400 cm^−1^ and used for qualitative peak assignment (lignin, cellulose/hemicellulose and related functional groups).

### 2.3. Experimental Design: Tequila Storage and Aging

Each container (plastic or oak) was filled to 90% of its nominal capacity with 55% (*v*/*v*) ethanol tequila and stored at ambient temperature (20 ± 5 °C) in a dark, ventilated room without agitation. The number of independent containers per material matched the availability in industry and our experimental design: one 250 L LDPE tank (n = 1), two 20 L HDPE jerry cans (n = 2), five 5 L PET carboys (n = 5), and two 225 L French oak barrels (one non-toasted, previously used for wine; one toasted, previously used for whisky; n = 1 each). Plastic containers were monitored for 18 months with monthly sampling; oak barrels were monitored for 11 months under identical conditions. At each sampling point, 50 mL of tequila was withdrawn, filtered, and stored at 4 °C until analysis.

Storage was performed at ambient temperature in a dark, ventilated room, using tequila at 55% ethanol and a 90% filling degree to standardize contact area and headspace across materials. Temperature, ethanol strength, headspace, and agitation are known drivers of migration; therefore, the reported kinetics are specific to these conditions and may differ under other storage/transport scenarios. In addition to temperature/ethanol/headspace, packaging geometry and wall thickness (affecting area-to-volume ratio and diffusion path length) as well as the initial DEHP content in the material can influence migration; we added container mass/thickness metadata and clarify that these drivers should be considered when extrapolating to other packaging designs.

### 2.4. DEHP Extraction and Quantification

DEHP extraction was performed using a liquid–liquid extraction technique in accordance with Gonzalez-Castro et al. [19]. A 10 mL aliquot of tequila was mixed with 5 mL of dichloromethane in a separatory funnel and vigorously shaken for 15 min twice. The organic phase was collected and dried, and then, the sample was resuspended in 1 mL of methanol. Gas Chromatography–Mass Spectrometry (GC–MS) analysis was carried out using an Agilent 7890B GC system coupled to a 5977A mass selective detector (Agilent Technologies, Santa Clara, CA, USA), equipped with a HP-5MS capillary column (30 m × 0.25 mm × 0.25 μm). The oven temperature was programmed as follows: initial temperature 60 °C (2 min hold), ramped to 300 °C at 10 °C/min (5 min hold). Helium was used as a carrier gas at a constant flow of 1.0 mL/min. The injection volume was 1 μL in splitless mode. Identification of DEHP was based on retention time and mass spectra compared to certified standards, with quantification performed using calibration curves in the range of 10–500 μg L^−1^. The injector (inlet) temperature was set to 250 °C.

### 2.5. Mass Transfer Models and Kinetic Fitting

To describe the migration kinetics of DEHP from each container type into the tequila matrix, the experimental data were fitted to:

1. Sorption/Desorption Model [20,21] (Exponential behavior)

$$C=\frac{t}{K_{1}+K_{2}t}$$

where

C: DEHP concentration (µg L^−1^) at time t (days)
*K*_1_ and *K*_2_: rate constants related to the extraction process, (L day µg^−1^ and L µg^−1^)
*t*: time (day)

2. Weibull-type Model [21,22] (Exponential behavior)

$$C=C_{0}e^{kt^{n}}$$

where

*C*: DEHP concentration (µg L^−1^) at time t (days)
*C*_0_: concentration at t = 0, (µg L^−1^)
*k*: reaction rate, (day^−1^)
*n*: reaction order
*t*: time, (day)

3. Minchev and Minkov Model [21,23] (Exponential behavior)

$$C=\alpha -\beta e^{-kt}$$

where

*C*: DEHP concentration (µg L^−1^) at time t (days)
*α*: saturation concentration, (µg L^−1^)
*β*: model constant, (µg L^−1^)
*k*: reaction rate, (day^−1^)
*t*: time, (day)

4. Two rates Model [21,24] (exponential model)

$$C=a\left[1-e^{-bt}\right]+c\left[1-e^{-dt}\right]$$

where

*C*: DEHP concentration (µg L^−1^) at time t (days)
*a*: concentration due to the extract, (µg L^−1^)
*b*: kinetic constant due to the extraction effect, (day^−1^)
*c*: concentration due to degradation, (µg L^−1^)
*d*: kinetic constant because of the degradation of the extract, (day^−1^)
*t*: time, (day)

5. Swelling/diffusion Model [21,25] (Exponential behavior)

$$C=\frac{C_{\infty }^{t}t}{T_{1/2}^{\infty }+t}+C_{\infty }^{d}\left[1-e^{-k_{d}t}\right]$$

where

*C*: DEHP concentration (µg L^−1^) at time t (days)
$C_{\infty }^{t}$: concentration of the extract at equilibrium, (µg L^−1^)
$C_{\infty }^{d}$: concentration of the extract at equilibrium due to diffusion, (µg L^−1^)
*k_d_*: kinetic constant due to the diffusion mechanism, (day^−1^)
$t_{1/2}^{\infty }$: half-life time, (day)
*t*: time, (day).

6. Second-order model [26] (Sigmoidal behavior)

$$C=C_{sat}\left[1-e^{-t/k}\left(1+\frac{t}{k}\right)\right]$$

where

*C*: DEHP concentration (µg L^−1^) at time t (days)
$C_{sat}:$ saturation concentration, (µg L^−1^)
k: kinetic constant, (day)
t: time, (day)

7. Modified Gompertz Model [27,28] (Sigmoidal behavior):

$$C=C_{max}\cdot exp\left[-exp\left(\frac{R_{max}\cdot e}{C_{max}}(\lambda -t)+1\right)\right]$$

where

*C*: DEHP concentration (µg L^−1^) at time t (days)
*C_max_*: asymptotic maximum concentration, (µg L^−1^)
*R_max_*: maximum transfer rate, (µg L^−1^ day^−1^)
*λ*: lag phase duration, (day)
*e*: Euler’s number
*t*: time, (day)

8. Fick’s Second Law Analytical Model [29]: (Deduction in Annex 1) The analytical solution for a semi-infinite slab was employed to determine the diffusion coefficient D of DEHP:

$$C_{1}\left(z,t\right)=C_{1\;0}+(C_{1\;\infty }-C_{1\;0})\cdot \left[erf\left(\frac{z}{2\sqrt{Dt}}\right)\right]$$

where

$C_{1}$*:* solute concentration at a given distance and time, (µg L^−1^)
$C_{1\;0}$*:* initial solute concentration in the surface layer (µg L^−1^)
$C_{1\;\infty }$*:* solute concentration at the end of the plate (µg L^−1^)
*z:* Distance traveled by the molecule (cm)
*D:* Diffusion coefficient (cm^2^ s^−1^)
*t:* Time, (s)

Model fitting and parameter estimation were conducted using nonlinear regression analysis in Excel Solver (Microsoft Excel, Microsoft 365, Version 16.0; Microsoft Corp., Redmond, WA, USA). The goodness-of-fit was evaluated using the coefficient of determination R2, root-mean-square error (RMSE), and Akaike Information Criterion (AIC). Model selection and inference: Because replication differs across container types (e.g., LDPE tank n = 1), between-material inferential statistics are interpreted cautiously. Model comparisons were therefore based primarily on objective goodness-of-fit metrics (e.g., RMSE, R2, and information criteria where applicable).

## 3. Results

### 3.1. Initial DEHP Levels and Background Contamination

The tequila utilized across all experimental conditions showed an initial DEHP concentration of 20.0 µg L^−1^, likely due to trace contamination from upstream industrial processes or temporary storage in plastic-lined tanks. Notably, the sample stored for one week in a previously used, non-toasted oak barrel (originally used for wine aging) exhibited a sharp increase to 88.25 µg L^−1^, indicating the barrel retained phthalates absorbed during prior use. This finding is consistent with previous studies showing that oak matrices can accumulate and later release phthalates during subsequent usage cycles. In contrast, the toasted oak barrel, which had been employed for whisky maturation, did not exhibit any meaningful increase in DEHP content, suggesting that thermal treatment significantly reduces phthalate migration potentially due to degradation of adsorbed phthalates and structural densification of the wood through charring [16].

### 3.2. Morphological and Chemical Characterization of Storage Materials

**Figure 1:**
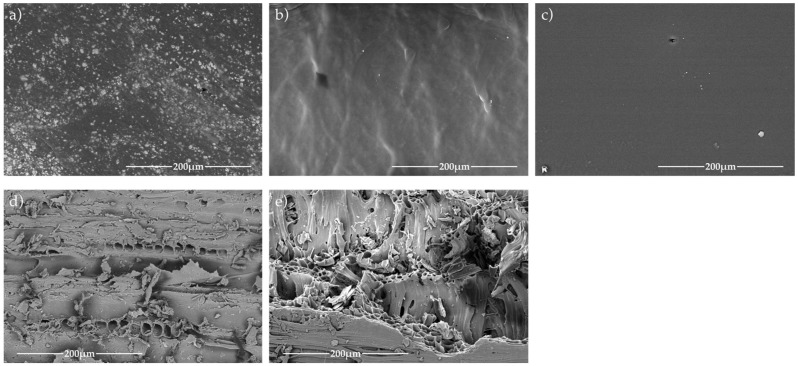
SEM micrographs of plastic and wood containers: (**a**) 250 L water tank, (**b**) 20 L jerry can, (**c**) 5 L carboy, (**d**) 230 L oak cask without thermal treatment, and (**e**) 230 L toasted oak cask.

SEM analysis revealed clear differences in surface topography between the plastic containers. PET and HDPE displayed smooth, homogeneous surfaces with minimal porosity, while the LDPE container exhibited irregular microstructures and shallow fissures, which may act as micro-reservoirs for phthalate release. These microstructural features likely influenced the migration rate observed during long-term storage (Figure 1). The comparatively rough appearance of some micrographs reflects real surface features (scratches, microvoids and pores) associated with manufacturing (e.g., rotational molding/blow molding), handling wear and, for wood, intrinsic porosity; these features are relevant because they increase interfacial area and can act as preferential pathways for sorption and diffusion.

These structural and chemical attributes suggest that LDPE containers may present the highest risk for phthalate migration, due to both higher permeability and surface heterogeneity. This is because LDPE has a higher amorphous fraction and greater free volume (lower crystallinity) than HDPE and PET, which generally increases sorption and diffusivity of hydrophobic plasticizers. In addition, the rougher, more heterogeneous surface observed by SEM increases the effective contact area and may provide microvoids or defects that facilitate mass transfer.

### 3.3. Time-Dependent Migration of DEHP in Plastic Containers

**Figure 2:**
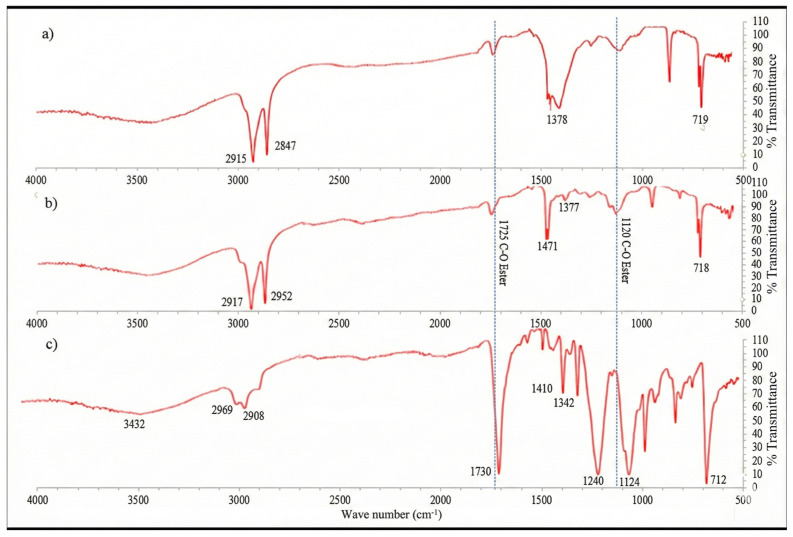
FTIR spectrum: (**a**) 250 L water tank, (**b**) 20 L jerry cans and (**c**) 5 L PET carboys (n = 5).

FTIR confirmed the identity of the polymers used, consistent with manufacturer specifications. The LDPE tank displayed characteristic C–H stretching bands near 2915 and 2848 cm^−1^. HDPE showed sharper, more intense peaks due to higher crystallinity. PET exhibited strong ester carbonyl absorption at 1715 cm^−1^ and aromatic C–O stretching at 1240 cm^−1^, confirming its polyester backbone (Figure 2).

Although the FTIR spectrum of DEHP presents several characteristic peaks, there are two that can help to presume its presence in plastic materials such as LDPE which lacks these bonds C=O (1725 cm^−1^) and C-O (1129 cm^−1^). Figure 2a–c exhibits the FTIR spectra of these containers, confirming their composition as LDPE in the water tank, HDPE in the jerry can and PET in the carboy, in addition to signals that suggest the presence of phthalates due to the presence of peaks in 1720 and 1120 cm^−1^.

**Figure 3:**
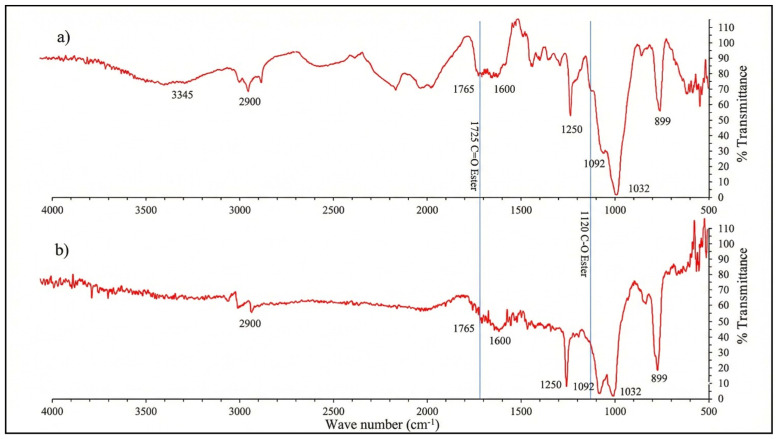
FTIR spectra of (**a**) oak cask without thermal treatment, (**b**) toasted oak cask.

In the FTIR spectra shown in Figure 3a,b, peaks associated with lignin and cellulose are identified, such as 3345 cm^−1^ attributed mainly to the hydroxyl groups, -OH; 1600 cm^−1^, due to C=C stretching and 1250 cm^−1^ attributed mainly to the asymmetric stretching of the carbonyl groups, and C=O [30]. Although signals appear in the characteristic region of phthalates (1720 and 1120 cm^−1^), they partially overlap with functional groups of wood, so their analytical confirmation was carried out by gas chromatography with mass spectrometry (Table 3).

### 3.4. Results of the Kinetic Study in Plastic Containers

**Figure 4:**
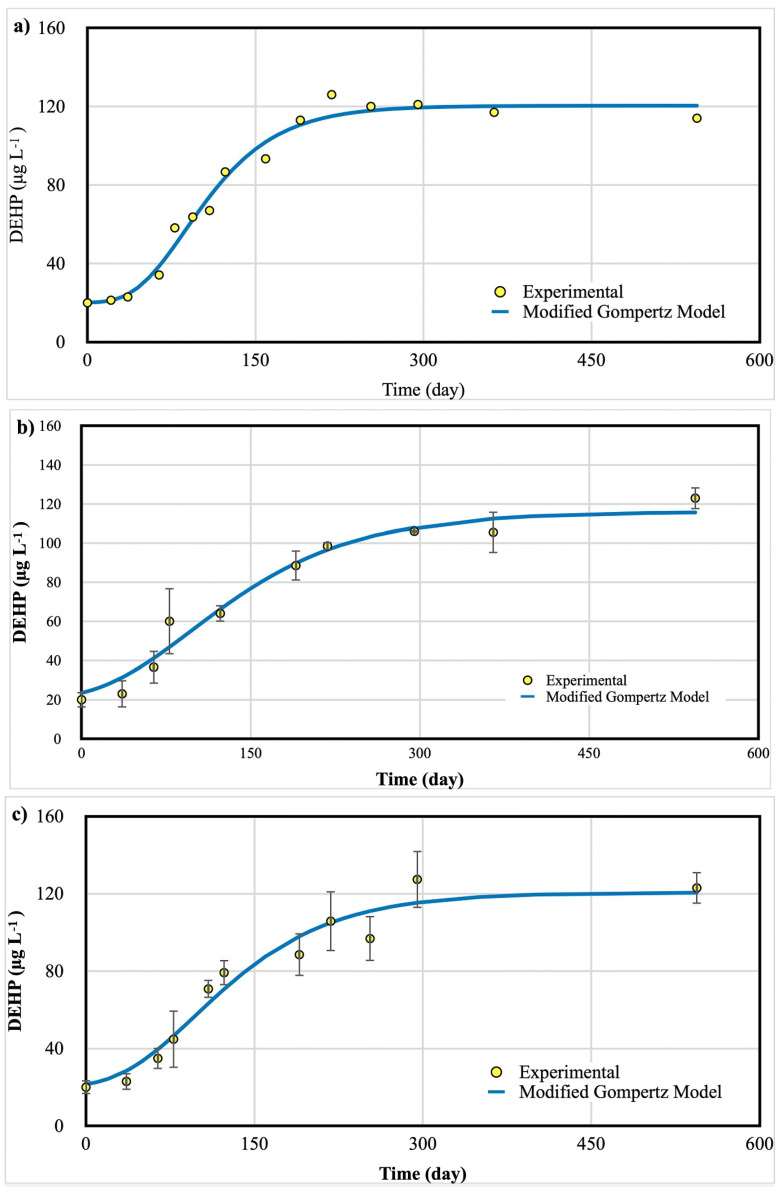
Migration of DEHP from plastic containers to tequila during 1.5 years of storage showed a sigmoidal-type fashion. (**a**) 250 L water tank (n = 1); (**b**) 20 L jerry cans (n = 2); (**c**) carboys (n = 5). Bars represent the standard deviation.

The kinetic parameters for the eight models in the four systems studied are shown in Table 4, Table 5 and Table 6. The DEHP concentrations in all plastic containers increased progressively over time, with dynamics best described by a sigmoidal pattern. The modified Gompertz model offered an optimal fit for each case (R^2^ > 0.97), capturing the lag phase, exponential acceleration, and final plateau as shown in Figure 4.

Minor non-monotonic fluctuations around the late-time plateau (including occasional small decreases) are consistent with analytical uncertainty and sampling variability rather than a true decline; the overall behavior is best interpreted as approaching an asymptote as captured by the fitted models.

The 250 L water tank exhibited the longest lag phase (~42 days), after which migration intensified rapidly. Final DEHP concentration plateaued at 118.2 µg L^−1^ by month 13. The prolonged lag phase is likely attributable to the sorption–desorption dynamics within LDPE’s amorphous domains. The 20 L jerry cans showed a shorter lag (~28 days) and a slightly lower maximum concentration (109.3 µg L^−1^), suggesting improved barrier properties compared to LDPE due to higher crystallinity and reduced permeability. Five L carboys demonstrated the lowest DEHP accumulation (88.7 µg L^−1^), with gradual but steady migration. This aligns with PET’s gas and contaminant barrier properties. These trends reinforce the material dependence of phthalate migration and suggest that PET may be more appropriate for long-term storage of ethanol-based beverages from a toxicological standpoint.

**Figure 5:**
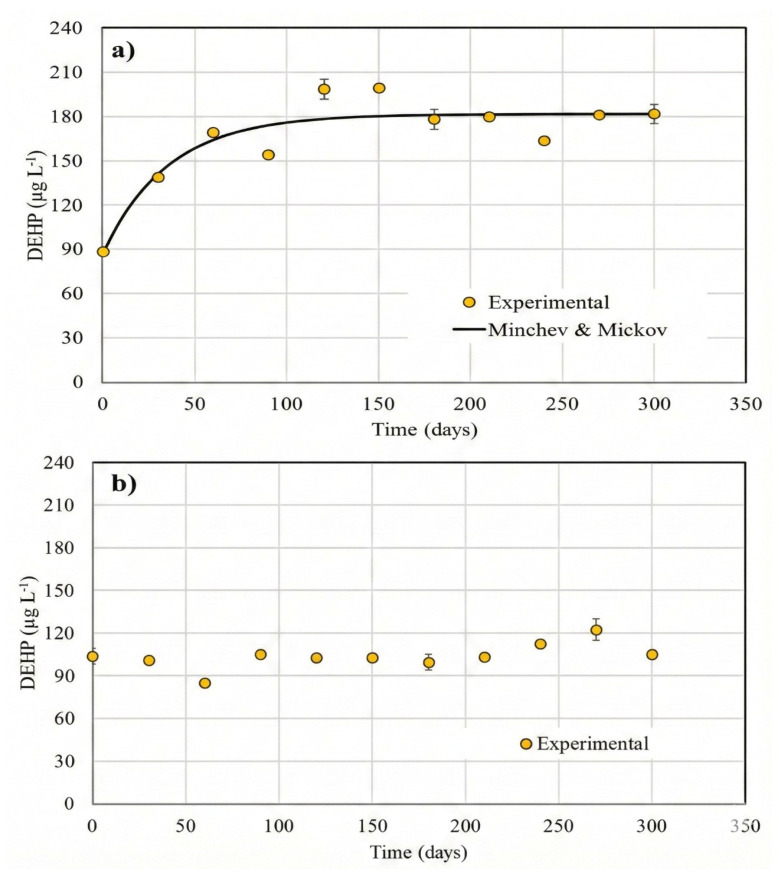
Migration of DEHP from oak cask to tequila for 10 months of aging. (**a**) Oak cask without thermal treatment. (**b**) Toasted oak cask.

The kinetic data were best fitted to the exponential Minchev and Minkov model (Figure 5), with a saturation concentration close to 185 µg L^−1^ and a rate constant (k) of 0.026 day^−1^.

Table 7 summarizes the kinetic coefficients of each model in the toasted and untoasted barrels, confirming that only in the latter case was there significant DEHP migration (Figure 5).

**Figure 6:**
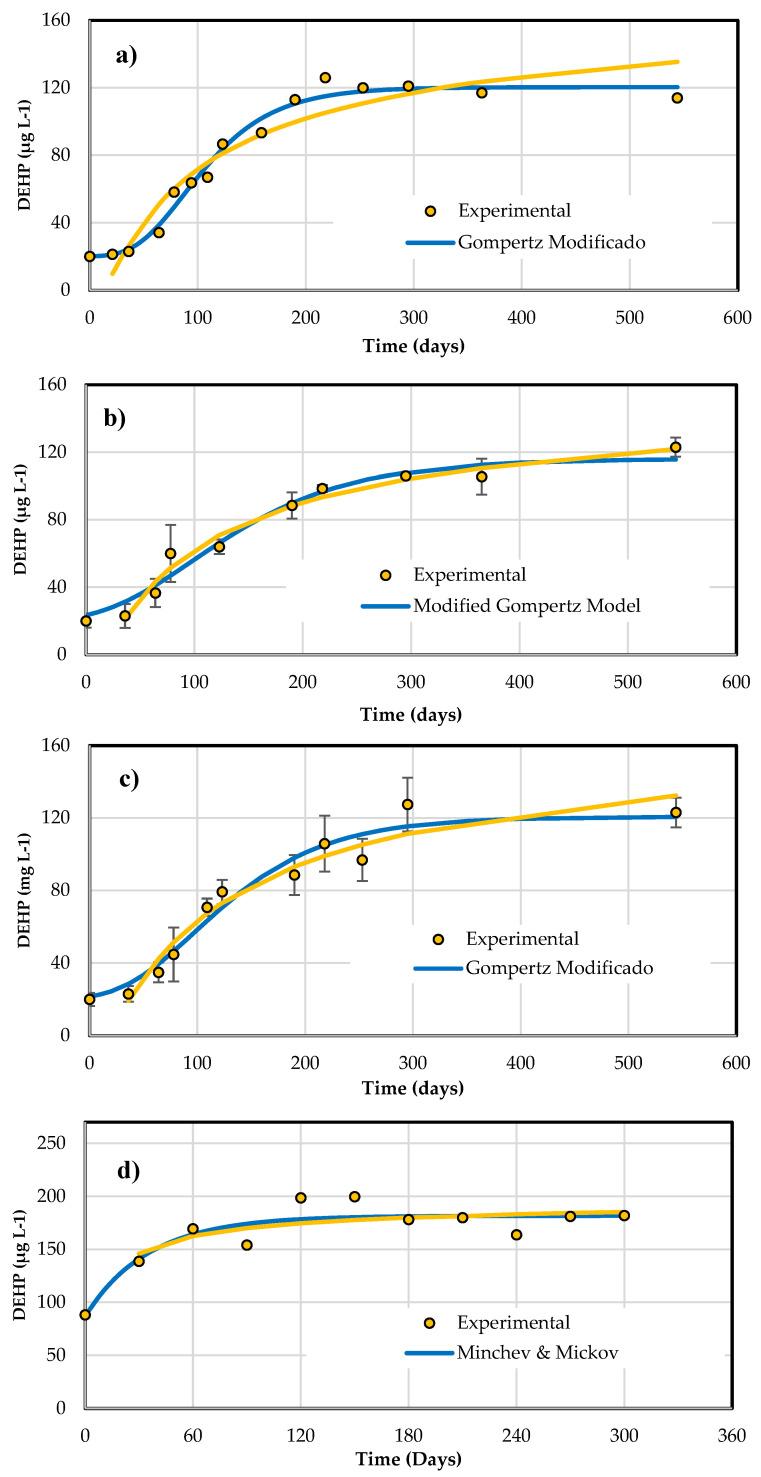
Projection of DEHP migration from plastic containers and oak cask into tequila, comparing empirical mass transfer models with the semi-infinite slab diffusion model derived from Fick’s second law for: (**a**) 250 L water tank, (**b**) 20 L jerry Cans, (**c**) 5 L carboys, and (**d**) oak cask without thermal treatment.

Figure 6 graphically shows the superposition of the best-fitting empirical curves with those of the semi-infinite plate diffusion model. This contrast highlights the need for a semi-empirical or empirical model when seeking to describe in detail the migration kinetics in matrices as heterogeneous as tequila and plastic materials, or wood with variable porosity.

### 3.5. Model Comparison and Estimation of Diffusion Coefficients

To gain mechanistic insights, all data sets were also fitted to an analytical model derived from Fick’s second law, assuming a semi-infinite slab geometry for migration (Figure 6). The estimated diffusion coefficients (D) varied depending on the container material: for 250 L water tank (LDPE), D = 1.21 × 10^−12^ m^2^ s^−1^; for 20 L jerry cans (HDPE), D = 8.74 × 10^−13^ m^2^ s^−1^; and for 5 L carboys (PET), D = 6.51 × 10^−13^ m^2^ s^−1^. These values are in line with prior work modeling plasticizer migration into aqueous alcohol solutions [9,31], and underscore the importance of container selection based on molecular transport properties.

Comparison of model performance using Akaike Information Criterion (AIC) and RMSE revealed that the Gompertz model outperformed others in capturing the sigmoidal behavior typical of plastic containers. The Minchev and Minkov model was better suited for the exponential migration seen in untreated wood, and Fickian diffusion modeling offered valuable estimation of intrinsic material transport properties.

## 4. Discussion

The results of this study reveal critical insights into the migration behavior of DEHP during long-term storage of tequila in different types of containers. While all plastic materials tested LDPE-, HDPE-, and PET-exhibited measurable DEHP release, the kinetics and final concentrations varied markedly based on polymer morphology, surface properties, and diffusion coefficients. The sigmoidal trends observed in plastic containers align with previous reports indicating that DEHP migration is governed by diffusion-controlled release followed by equilibrium partitioning with the food simulant or beverage matrix. The pronounced lag phase in LDPE tanks, followed by a rapid increase in DEHP levels, underscores the importance of molecular mobility within amorphous domains of semicrystalline polymers. In contrast, PET’s lower diffusivity and higher crystallinity resulted in reduced and slower migration, confirming its superior barrier properties [7,10]. These results highlight PET’s relative safety when compared with other food-grade plastics for high-ethanol content beverages, although even PET cannot fully eliminate contaminant migration over long durations. Materials are compared under the same experimental conditions (55% ethanol, ambient storage, 90% fill) and differing replication across containers limits inferential claims; thus, conclusions are scope-limited to the tested items and conditions.

The behavior of oak barrels revealed an equally compelling narrative. The untreated oak cask previously used for wine storage released residual DEHP to a greater extent than any plastic container, suggesting that phthalates previously sorbed into the wood’s porous network may be re-extracted by ethanol solutions. This phenomenon is especially pervasive concerning alcoholic beverages, given ethanol’s high solvating capacity and its ability to penetrate wood microstructures. In stark contrast, the toasted oak barrel used for whisky aging exhibited minimal DEHP release, supporting prior observations that thermal degradation and charring reduce extractive migration potential by altering wood porosity and degrading residual contaminants. We clarify that quantifying DEHP in wood prior to the migration test is less straightforward than in homogeneous polymers due to wood heterogeneity and layered sorption history; we therefore treat this as a limitation and a priority for future work (targeted extraction of surface shavings/extractives).

From a regulatory standpoint, the highest observed DEHP concentration (185.6 µg L^−1^ in the untreated oak barrel) is below the maximum levels reported in international guidance for non-fat foods and beverages (e.g., 1500 µg kg^−1^ for DEHP). For comparison, using a typical density for 55% (*v*/*v*) ethanol solutions (~0.93 kg L^−1^), 185.6 µg L^−1^ corresponds to ~200 µg kg^−1^. Nevertheless, the gradual accumulation observed over months of storage—even in PET containers—indicates that prolonged contact between high-ethanol beverages and certain materials can lead to cumulative contamination, particularly when storage times exceed six months. Regulatory comparisons were derived from primary regulatory documents and official guidance (e.g., EFSA and other competent authorities), rather than secondary web overviews.

Importantly, the kinetic models applied in this work provide valuable tools not only for describing empirical behavior but also for predicting long-term exposure scenarios. The modified Gompertz model allowed for accurate prediction of DEHP saturation points, which could inform shelf-life estimations and risk assessments for products stored in plastic containers. Meanwhile, the analytical solution based on Fick’s second law enabled calculation of diffusion coefficients, contributing to the mechanistic understanding of phthalate transfer in complex liquid–solid systems.

These findings bear significant implications for the tequila industry, where plastic containers are commonly used for bulk storage and transport, and oak barrels are employed for premium aging processes. Based on data generated in this study, several practical recommendations emerge: avoid long-term storage (>6 months) in LDPE or HDPE containers unless barriers or liners are applied, favor PET containers for ethanol-based beverage storage when plastic use is unavoidable, perform thermal treatment (toasting or charring) on reused oak barrels to reduce contaminant release, and regularly monitor DEHP levels in aged products, especially those destined for export markets with stricter phthalate regulations.

Beyond tequila, this study contributes broadly to the understanding of plasticizer migration in alcoholic beverages, a topic of growing concern in the global food safety landscape. While this study focused on DEHP, similar approaches could be extended to other phthalates and emerging contaminants such as bisphenols and microplastics. Citations in this section refer specifically to diffusion/partitioning in bulk polymers and migration testing in alcoholic matrices (rather than microplastics), and the reference list has been adjusted accordingly.

## 5. Conclusions

From an industrial and regulatory standpoint, these findings underscore the importance of container selection and treatment history in ensuring product safety during beverage aging. Although observed DEHP levels remained below international regulatory thresholds, the data highlight the potential for cumulative contamination during long-term storage, particularly when using low-barrier plastics and untreated wooden barrels. Future prospects include: (i) extending the approach to additional plasticizers and emerging migrants (bisphenols, oligomers, microplastics) and to other spirits and ethanol strengths; (ii) quantifying temperature dependence and real supply-chain scenarios to build accelerated, yet mechanistically consistent, shelf-life tests; (iii) validating mitigation strategies such as barrier liners/coatings, material selection guidelines and reuse/conditioning protocols for barrels; and (iv) integrating the best-performing kinetic models into routine QC tools for conservative forecasting and decision-making.

## Figures and Tables

**Table 1:** Plastic containers used for storage of tequila in small and medium-sized production factories.

Type of Container	Volume	Composition	Used for (Storage Time)
Cisterns	5000–15,000 L	LDPE	Long-term storage (Two years)
Water tanks	250–2500 L	LLDPE	Long-term and short-term storage (Two years)
Square containers	1000 L	HDPE	Transportation and short-term storage (One year)
Water drums	230 L	HDPE	Transportation and short-term storage (One year)
Jerry cans	10–60 L	HDPE or LDHP	Short-term storage and wholesales (One year)
Carboys	1–10 L	PET	Retail sales (Two years)

LDPE: Low-Density Polyethylene; LLDPE: Linear Low-Density Polyethylene; HDPE: High-Density Polyethylene; PET: Polyethylene Terephthalate.

**Table 2:** Phthalates concentrations permitted by international standards to non-fat meals, including distilled alcoholic beverages [10,11,12,13].

Abbreviation	Name	Structure	Permitted Limit (µg kg^−1^)
DBP	Di-butyl phthalate	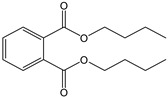	300
DEHP	Di-ethyl-hexyl phthalate	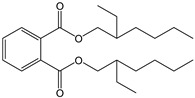	1500
DINP	Di-isononyl phthalate	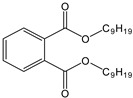	9000

**Table 3:** Plastic containers used during the tequila storage experiments.

Type of Container	Number of Assays	Composition	Vol. of Tequila (L)	Volume/Contact Area (L m^−2^)	DEHP (mg kg^−1^)
250 L Water tank (Rotoplas^®^, Monterrey, NL, Mexico)	1	LDPE	170	130.9	42.6
20 L Jerry cans (Visapack^®^, Tlanepantla, Edo. de Mex., Mexico)	2	HDPE	20	38.7	81.3
5 L Carboys (Jalmex^®^, Guadalajara, Jal., Mexico)	5	PET	5	25.1	283.8

LDPE: Low-Density Polyethylene; HDPE: High-Density Polyethylene; PET: Polyethylene Terephthalate. Note: DEHP-in-polymer values are reported as mean ± SD of replicate extractions/analyses (where applicable).

**Table 4:** Kinetic coefficients of DEHP migration from 250 L water tank to tequila.

Model Name	Kinetic Parameter Values (Units in 2.5)			Model Evaluation Parameters	
Sorption/Desorption	K_1_ = 1.320	K_2_ = 0.006			R^2^ = 0.929 *p* = 2.510 × 10^−8^
Weibull-type	C_0_ = 1.501 × 10^−6^	k = 15.027	n = 0.0308		R^2^ = 0.8230 *p* = 5.991 × 10^−5^
Minchev & Minkov	α = 114.999	β = 114.99	k = 0.006		R^2^ = 0.962 *p* = 1.660 × 10^−4^
Two-rate	a = 57.498	b = 0.006	c = 57.498	d = 0.006	R^2^ = 0.962 *p* = 7.788 × 10^−9^
Swelling/Diffusion	C^w^_∞_ = 162.730	T^w^_1/2_ = 214.7	C^d^_∞_ = 0.00	k_d_ = 1.00	R^2^ = 0.929 *p* = 5.867 × 10^−7^
Second-order	C_sat_ = 105.254	k = 62.904			R^2^ = 0.997 *p* = 1.334 × 10^−17^
Modified Gompertz	C_max_ = 100.384	R_max_ = 0.820	λ = 42.368		R^2^ = 0.9829 *p* = 6.905 × 10^−12^
Fick’s Second Law Analytical Model		D = 1.21 × 10^−12^ m^2^ s^−1^			R^2^ = 0.9057 *p* = 1.532 × 10^−5^

**Table 5:** Kinetic coefficients of DEHP migration from 20 L jerry cans to tequila.

Model Name	Kinetic Parameter Values (Units in 2.5)			Model Evaluation Parameters	
Sorption/Desorption	K_1_ = 1.873	K_2_ = 0.006			R^2^ = 0.9926 *p* = 7.877 × 10^−10^
Weibull-type	C_0_ = 6.828 × 10^−6^	k = 13.312	n = 0.0349		R^2^ = 0.8728 *p* = 1.048 × 10^−3^
Minchev & Minkov	α = 115.167	β = 115.167	k = 0.004		R^2^ = 0.9997 *p* = 4.147 × 10^−15^
Two-rate	a = 0.000	b = 1.000	c = 115.070	d = 0.004	R^2^ = 0.9996 *p* = 1.965 × 10^−14^
Swelling/Diffusion	C^w^_∞_ = 168.723	T^w^_1/2_ = 316.03	C^d^ = 0.00	k_d_ = 1.00	R^2^ = 0.9926 *p* = 1.920 × 10^−8^
Second-order	C_sat_ = 96.798	k = 74.686			R^2^ = 0.9762 *p* = 1.478 × 10^−7^
Modified Gompertz	C_max_ = 96.088	R_max_ = 0.435	λ = 16.478		R^2^ = 0.9907 *p* = 1.141 × 10^−8^
Fick’s Second Law Analytical Model		D = 8.74 × 10^−13^ m^2^ s^−1^			R^2^ = 0.9076 *p* = 1.728 × 10^−4^

**Table 6:** Kinetic coefficients of DEHP migration from 5 L carboys to tequila.

Model Name	Kinetic Parameter Values (Units in 2.5)			Model Evaluation Parameters	
Sorption/Desorption	K_1_ = 1.721	K_2_ = 0.005			R^2^ = 0.960 *p* = 3.160 × 10^−7^
Weibull-type	C_0_ = 7.095 × 10^−6^	k = 13.346	n = 0.0349		R^2^ = 0.847 *p* = 8.846 × 10^−4^
Minchev & Minkov	α = 123.479	β = 123.479	k = 0.004		R^2^ = 0.978 *p* = 8.476 × 10^−8^
Two-rate	a = 61.741	b = 0.004	c = 61.741	d = 0.004	R^2^ = 0.978 *p* = 3.855 × 10^−7^
Swelling/Diffusion	C^w^_∞_ = 182.646	T^w^_1/2_ = 314.38	C^d^_∞_ = 0.00	k_d_ = 1.00	R^2^ = 0.960 *p* = 6.947 × 10^−6^
Second-order	C_sat_ = 105.789	k = 76.162			R^2^ = 0.981 *p* = 7.231 × 10^−9^
Modified Gompertz	C_max_ = 100.701	R_max_ = 0.546	λ = 29.673		R^2^ = 0.974 *p* = 1.926 × 10^−7^
Fick’s Second Law Analytical Model		D = 6.51 × 10^−13^ m^2^ s^−1^			R^2^ = 0.945 *p* = 1.732 × 10^−4^

**Table 7:** Kinetic coefficients of DEHP migration from oak cask without thermal treatment to tequila.

Model Name	Kinetic Parameter Values (Units in 2.5)			Model Evaluation Parameters	
Sorption/Desorption	K_1_ = 0.239	K_2_ = 0.009			R^2^ = 0.894 *p* = 3.533 × 10^−5^
Weibull-type	C_0_ = 88.250	k = 0.328	n = 0.0134		R^2^ = 0.843 *p* = 1.006 × 10^−3^
Minchev & Minkov	α = 97.007	β = 97.663	k = 0.026		R^2^ = 0.897 *p* = 1.390 × 10^−4^
Two-rate	a = 96.632	b = 0.026	c = 0.3915	d = 0.0259	R^2^ = 0.909 *p* = 3.096 × 10^−4^
Swelling/Diffusion	C^w^_∞_ = 108.531	T^w^_1/2_ = 25.993	C^d^_∞_ = 0.001	k_d_ = 5.711	R^2^ = 0.894 *p* = 6.250 × 10^−4^
Second-order	C_sat_ = 92.410	k = 17.446			R^2^ = 0.822 *p* = 4.342 × 10^−4^
Modified Gompertz	C_max_ = 92.349	R_max_ = 1.678	λ = 1.656		R^2^ = 0.899 *p* = 2.741 × 10^−5^
Fick’s Second Law Analytical Model			Not reported (poor fit)		R^2^ = 0.430

## Data Availability

The original contributions presented in this study are included in the article/Supplementary Materials. Further inquiries can be directed to the corresponding authors.

## Supplementary Materials

The following supporting information can be downloaded at: https://www.mdpi.com/article/10.3390/foods15081380/s1, Document: Diffusion in a Semi-Infinite Slab.
