# Supplementary material for: Migration of Diethyl-Hexyl-Phthalate from Plastic Containers and Oak Casks to Tequila During Long-Term Storage and Aging
Source: Foods. 2026 Apr 15;15(8):1380. doi: 10.3390/foods15081380 (PMC13114747; doi:10.3390/foods15081380)
Supplement: Supplementary file 1 [file foods-15-01380-s001.zip › foods-4165315-supplementary.pdf]

SUPPLEMENTARY MATERIAL

Diffusion in a Semi-Infinite Slab [29]

Physical Situation

The mass transfer phenomenon is considered to occur in a semi-infinite slab, where the initial solute concentration is uniform and denoted as $c_{1\infty}$. At time $t = 0$, the concentration at the interface $z = 0$ is abruptly raised to a new value c_{10}. The goal is to determine how the solute concentration c_1 changes as a function of position z and time t.

Mathematical Formulation

1 Mass Balance Equation

The mass balance over a differential control volume $A\Delta z$, where A is the cross-sectional area and Δz is the differential length, is:

Accumulation = Inflow by diffusion - Outflow by diffusion

Mathematically:

$$\frac{\partial}{\partial t}(A\Delta z c_1) = A[j_1|_z - j_1|_{z+\Delta z}]$$

Dividing both sides by $A\Delta z$:

$$\frac{\partial c_1}{\partial t} = \frac{j_1|_z - j_1|_{z+\Delta z}}{\Delta z}$$

Taking the limit as $\Delta z \to 0$, we obtain:

$$\frac{\partial c_1}{\partial t} = -\frac{\partial j_1}{\partial z}$$

2 Fick's Law of Diffusion

The diffusive flux is governed by Fick's first law:

$$j_1 = -D\frac{\partial c_1}{\partial z}$$

Assuming a dilute system and constant diffusion dominated by mass transfer, substitution into the mass balance yields:

$$\frac{\partial c_1}{\partial t} = D\frac{\partial^2 c_1}{\partial z^2}$$

Initial and Boundary Conditions

- At $t = 0$, for all z :

$$c_1 = c_{1\infty}$$

Initially, the solute is absent from the alcoholic solution; hence, it is uniformly distributed in the plastic matrix.

- At $t > 0$, at $z = 0$:

$$c_1 = c_{10}$$

As diffusion starts, solute begins migrating from the plastic into the adjacent liquid layer.

At $t > 0$, as $z \to \infty$:

$$c_1 = c_{1\infty}$$

At sufficient distance from the surface, concentration remains unchanged at initial value.

Solution to the Diffusion Equation

We define the dimensionless variable:

$$\zeta = \frac{z}{2\sqrt{Dt}}$$

Where:

- z is position in the slab
- D is the diffusion coefficient
- t is time

This transformation converts the partial differential equation into an ordinary differential equation (ODE), facilitating its solution.

Chain Rule Derivatives

- Temporal derivative:
- Temporal derivative:

$$\frac{\partial c_1}{\partial t} = \frac{\partial c_1}{\partial \zeta} \cdot \frac{\partial \zeta}{\partial t} = \frac{\partial c_1}{\partial \zeta} \cdot \left(-\frac{\zeta}{2t}\right)$$

- First spatial derivative:

$$\frac{\partial c_1}{\partial z} = \frac{\partial c_1}{\partial \zeta} \cdot \frac{1}{2\sqrt{Dt}}$$

- Second spatial derivative:

$$\frac{\partial^2 c_1}{\partial z^2} = \frac{1}{4Dt} \cdot \frac{\partial^2 c_1}{\partial \zeta^2}$$

Substituting into the diffusion equation:

$$-\frac{\zeta}{2t} \cdot \frac{\partial c_1}{\partial \zeta} = D \cdot \frac{1}{4Dt} \cdot \frac{\partial^2 c_1}{\partial \zeta^2} \Rightarrow 2\zeta \frac{\partial c_1}{\partial \zeta} = -\frac{\partial^2 c_1}{\partial \zeta^2}$$

Rewriting:

$$\frac{\partial^2 c_1}{\partial \zeta^2} + 2\zeta \frac{\partial c_1}{\partial \zeta} = 0$$

Solution Using Error Function

The general solution of the ODE is:

$$\frac{\partial c_1}{\partial \zeta} = ae^{-\zeta^2} \Rightarrow c_1 = \int ae^{-\zeta^2} d\zeta$$

Using the error function:

$$\mathrm{erf}\,(\zeta) = \frac{2}{\sqrt{\pi}} \int_0^{\zeta} e^{-s^2} ds$$

The full solution becomes:

$$c_1(z,t) = c_{10} + (c_{1\infty} - c_{10}) \cdot \mathrm{erf}\,\left(\frac{z}{2\sqrt{Dt}}\right)$$

Approximate Series Solution

A series approximation of the error function yields:

$$c_1(z,t) \approx c_0 \left[1 - \frac{2}{\sqrt{\pi}}\left(\frac{z}{2\sqrt{Dt}} - \frac{1}{3}\left(\frac{z}{2\sqrt{Dt}}\right)^3 + \frac{1}{10}\left(\frac{z}{2\sqrt{Dt}}\right)^5 - \frac{1}{42}\left(\frac{z}{2\sqrt{Dt}}\right)^7\right)\right]$$

Diffusive Flux

The instantaneous flux from the surface is obtained using Fick's law:

$$j_1 = -D \cdot \frac{\partial c_1}{\partial z}$$

Substituting the solution:

$$j_1 = -D \cdot \frac{\partial}{\partial z}\left[c_{10} + (c_{1\infty} - c_{10}) \cdot \mathrm{erf}\,\left(\frac{z}{2\sqrt{Dt}}\right)\right]$$

Using the derivative of the error function:

$$\frac{\partial}{\partial z}\mathrm{erf}\,\left(\frac{z}{2\sqrt{Dt}}\right) = \frac{1}{\sqrt{\pi Dt}} e^{-z^2/4Dt}$$

Thus, the flux becomes:

$$j_1 = -D(c_{1\infty} - c_{10}) \cdot \frac{1}{\sqrt{\pi Dt}} e^{-z^2/4Dt}$$

At the interface $z = 0$:

$$j_1|_{z=0} = \frac{D}{\sqrt{\pi t}}(c_{10} - c_{1\infty})$$
